# Isolation and Characterization of Microsatellite Markers in the Domestic Ferret (*Mustela putorius furo*)

**DOI:** 10.3390/ijms131216592

**Published:** 2012-12-05

**Authors:** Holly B. Ernest, Tracy L. Drazenovich, Lisa S. Dalbeck, Michelle G. Hawkins

**Affiliations:** 1 Wildlife Ecology Unit, Veterinary Genetics Laboratory, School of Veterinary Medicine, One Shields Ave, Davis, CA 95616, USA; E-Mail: ldalbeck@ucdavis.edu; 2 Department of Population Health and Reproduction, School of Veterinary Medicine, One Shields Ave, Davis, CA 95616, USA; 3 Department of Medicine and Epidemiology, School of Veterinary Medicine, One Shields Ave, Davis, CA 95616, USA; E-Mails: tldraz@ucdavis.edu (T.L.D.); mghawkins@ucdavis.edu (M.G.H.)

**Keywords:** *Mustela putorius furo*, domestic ferret, microsatellite, polymorphism, genetic diversity, multiplex panel

## Abstract

The domestic ferret (*Mustela putorius furo*) is an important model organism for the study of avian influenza and other diseases of humans and animals, as well as a popular pet animal. In order to evaluate genetic diversity and study disease relationships in ferrets, 22 nuclear microsatellite loci (17 dinucleotide and 5 tetranucleotide) were developed from ferret genomic libraries and organized into seven multiplex sets. Polymorphism was preliminarily assessed in one population in Australia and one in the USA, sampled with 25 individuals each. The loci displayed allelic diversity ranging from 1 to 5 alleles, and expected and observed heterozygosities ranging from 0.04 to 0.65 and 0.04 to 0.76, respectively. Additionally, the loci amplified products in 15 samples from the wild ancestor, European polecat (*Mustela putorius*) and domestic ferret-polecat hybrids. In polecat/hybrid samples, allelic diversity ranged from 3 to 8 alleles, and expected and observed heterozygosities ranged from 0.13 to 0.81 and 0.13 to 0.80 respectively. These markers will be useful for molecular assessments of genetic diversity and applications to evolution, ecology, and health in domestic ferrets and wild polecats.

## 1. Introduction

The domestic ferret (*Mustela putorius furo*) is used as a model organism for the study of disease states important to human health including avian influenza [1], immune response of enteric bacterial pathogens (*Campylobacter jejuni*) [2] and morbidity associated with obesity [3], to name a few. They are increasingly popular as house pets throughout many parts of the world. The domestic ferret was first domesticated several thousand years ago from the ancestral wild mustelid species, European Polecat (*Mustela putorius*) [4].

In order to provide molecular tools for genetic assessments at the genome, individual and population level, we identified 22 polymorphic nuclear microsatellite regions in the domestic ferret genome, then developed and optimized multiplex groups of PCR primers to efficiently provide polymorphic genotypic data.

## 2. Results and Discussion

A total of 126 microsatellite sequences were isolated from partial genome libraries using the protocol of Jones *et al*. [5]. Forty-eight were excluded from further consideration due to presence of ambiguous reads on electropherograms or flanking regions with runs of single nucleotide repeats. The remaining 78 sequences consistently yielded clear DNA bands in domestic ferrets from Australia, the USA, as well as wild polecats and hybrids between polecat and domestic ferrets. The final 22-marker set displayed polymorphism and amplification products within the expected size range (Table 1). In domestic ferrets, the markers did not display significant departures from expectations of Hardy-Weinberg and linkage equilibria (Table 2).

In 25 domestic ferrets tested from Australia, three loci were monomorphic (mpuA212w, mpuB1w, and mpuC102w), and alleles per locus ranged from 1–5, with an average of 2.3 alleles per locus for the 22 markers. Expected (*H*_E_) and observed (*H*_O_) heterozygosities ranged from 0.11 to 0.76 with averages of 0.37 (*H*_E_) and 0.35 (*H*_O_). In 25 domestic ferrets tested from USA, three loci were monomorphic (mpuA115w, mpuA129w, mpuC102w), and alleles per locus ranged from 1–4, with an average of 2.2 alleles per locus. Heterozygosities ranged from 0.04 to 0.68 with averages of 0.31 (*H*_E_) and 0.31 (*H*_O_).

A third set of 15 samples contained European polecats (*Mustela putorius*, *N* = 6) and hybrids of *Mustela putorius furo* plus one of the following at 50% or more in reported pedigree: *Mustela putorius* (*N* = 4), *Mustela eversmanni* (*N* = 4), *and Mustela siberica* (*N* = 1). In the polecats/hybrids set, no loci were monomorphic, and alleles per locus ranged from 3 to 8, with an average of 4.8 alleles per locus. Heterozygosities ranged from 0.13–0.81 with averages of 0.56 (*H*_E_) and 0.49 (*H*_O_). None of the loci tested in the two domestic ferret populations deviated significantly from expectations of Hardy-Weinberg equilibrium with Bonferroni correction applied, and there were no indications of null alleles.

## 3. Experimental Section

Four libraries enriched with microsatellite motifs (CA, GA, CATC, and TAGA) were created by Genetic Identification Services, Inc. (Chatsworth, CA, USA) with DNA extracted from a female domestic ferret following the methods of Jones *et al*. [5]. Of 126 sequences containing potential microsatellite regions 48 were excluded from further consideration due to presence of ambiguous reads on electropherograms or flanking regions with runs of single nucleotide repeats. The remaining 78 sequences screened for polymorphism and PCR amplification quality. Primer3 (version 0.27) software was used to design the PCR primers. These primers were initially screened with universal M13 tails as in Schuelke’s protocol [7]. The reverse primers were designed with a 5′ PIG-tail (GTTCTT) to facilitate adenylation [6].

DNA was extracted from oral swab or plucked hair. Hair samples were collected from the base of the tail and stored at room temperature until DNA extraction. Bulbs of 4–10 plucked hairs from each ferret were incubated with 100 μL of hair lysis buffer and 0.5 μL proteinase K for 45 min at 60 °C, then 45 min at 95 °C. Each 100 μL of hair lysis buffer contained 83 μL water, 8.3 μL 10× PCR buffer, 8.3 μL 25 mM MgCl_2_, and 0.4 μL Tween 20. The samples were quickly spun down and the resulting solution was used for PCR. If no hair was collected, oral swabs were used following protocols adapted from the French National Institute for Agricultural Research. The swabs were placed in 200 μL of 200 mM NaOH solution and incubated for 10 min at 95 °C. Next swabs were removed and 200 μL of 1 M Tris-HCl solution was added. Samples were stored at 4 °C for immediate use or −20 °C for future use.

Primers that had no more than two alleles per individual, products of expected size and polymorphism, were traditionally fluorescently dye labeled (NED, PET, FAM or VIC; ABI) and optimized in multiplex groups based on base pair size and fluorescent dye compatibility. Two μL of DNA was used with a QIAGEN Multiplex PCR Kit (QIAGEN Inc., Valencia, CA, USA). Volumes of each reagent were reduced from Multiplex kit instructions to 6.25 μL of 2× QIAGEN multiplex PCR master mix (final concentration 1×), 1.25 μL of primer mix (final concentration of 0.03 to 0.05 μM for each primer), 1.25 μL Q solution, 1.75 μL of distilled water (3 μL if no Q solution), and 2 μL of DNA for a total volume of 12.5 μL.

Amplifications were carried out in a Bio-Rad MyCyler (Bio-Rad, Hercules, CA, USA) using the Multiplex PCR protocol for amplification of microsatellite loci with and without Q solution (QIAGEN Multiplex PCR kit; QIAGEN). All samples were initially run through the protocol for only 10 cycles. Two μL of that product was then used to make new sample for PCR that was then run for the suggested cycle times. For the multiplexes that used Q solution: 15 min at 95 °C (initial activation step), followed by 40 cycles consisting of 94 °C for 30 s, 60 °C for 90 s, and 72 °C for 90 s (except for multiplex 6 whose middle step was 58 °C). The last extension step was at 72 °C for 10 min. For the multiplexes that did not use Q solution: 15 min at 95 °C (initial activation step), followed by 35 cycles consisting of 94 °C for 30 s, 60 °C for 90 s, and 72 °C for 60 s. The last extension step was at 60 °C for 30 min.

PCR products were separated with an ABI PRISM 3730 DNA Analyzer (Applied Biosystems Inc. Foster City, CA, USA) with each capillary containing 1 μL of a 1:10 dilution of PCR product and deionized water, 0.05 μL GeneScan-500 LIZ Size Standard and 9.95 μL of HiDi formamide (both products Applied Biosystems Inc.) that was denatured at 95 °C for 3 min. Products were visualized with STRand version 2.3.69 [8].

Genetic diversity was determined for 25 domestic ferrets from both Australia and USA. An additional 15 wild polecats/hybrids were analyzed. For each locus, the number of alleles (N_a_), observed heterozygosity (*H*_O_), expected heterozygosity (*H*_E_), tests for linkage disequilibrium and deviations from Hardy-Weinberg equilibrium were calculated using software GenAlEx [9].

## 4. Conclusions

To our knowledge the markers of this study represent the first published microsatellite markers developed specifically from domestic ferret DNA. These loci will be useful for molecular genetics assessments of genetic diversity and applications to evolution, ecology, and health in domestic ferrets and wild polecats.

## Figures and Tables

**Table 1:** Characterization of the 22 microsatellite loci developed for domestic ferrets, *Mustela putorius furo*. All reverse primers had a PIG-tail added (gttctt) [6]. The multiplex groups (noted in table by superscripts) 1, 3, 4, 6, and 7 required Q solution contained in the QIAGEN Multiplex PCR Kit. The multiplex groups 2 and 5 did not require Q solution.

Locus	Dye label	PCR product size (bp)	Repeat motif	Primer sequence (5′-3′)	GenBank Accession no.
Mpu A4w ^1^	vic	152–174	(AC)23	F: CACTTCCTCCCATGGACACT R: CAAAGTCTCCCACCCTATGC	JX469575
Mpu A10w	ned	154–158	(AC)13(AC)7	F: TGGCCTATATGTGCAGATGAC R: TGTTTGTCTTGTACCCTCTGACC	JX469576
Mpu A115w	pet	164–188	(TG)17	F: CACTGTTAGGGGAAGGAAGA R: CTAAGAGTGTTCCTGATTGCAT	JX469577
Mpu A121w ^7^	fam	105–129	(CA)14	F: ACTGCCATCAGGTCATCTAGG R: GGGTAGACACCTGGCTCAAG	JX469578
Mpu A129w ^7^	fam	197–205	(CA)13	F: GGCCTCTGAACACATAGTTG R: AAGTACAGAATGGAAGGATCTG	JX469579
Mpu A212w ^1^	fam	137–153	(TG)13	F: CCCTATGAGGGCATGTTTGT R: CTGCCATGTTTCCACTGGT	JX469580
Mpu A223w ^3^	ned	213–239	(GT)19	F: GAAGACAGCACCCCAGAGTC R: TGGTTGCCAAGAACTAGCAG	JX469581
Mpu A229w ^4^	pet	107–139	(GT)11(TG)7	F: GGGTAGGACGTGCTTAAAGATG R: AGCCCTCAAAGCCTCTTCTC	JX469582
Mpu A231w ^6^	vic	182–224	(GT)15	F: CCTCTGGTAACCATCTGTTTG R: TCTTCAAGATGTTCAGTGTGGA	JX469583
Mpu B1w ^4^	vic	178–190	(CT)15(CA)8 (AC)5(AC)11	F: TCCACTACCTGGCCTCATTC R: ACCTCAGGCTCCACTCTCAG	JX469584
Mpu B6w ^2^	ned	156–164	(TC)18	F: TGGGTGTAGAGCATGTTTGG R: TGCCTATTCCAGGTACCTCAT	JX469585
Mpu B9w ^7^	vic	180–192	(GA)18	F: CGTTACCAACTGTGGCTGTG R: TGCCTGGGCCTGTGATTA	JX469586
Mpu B12w ^3^	fam	175–179	(AG)11(AC)8 (AC)7	F: AGTCGACAGATGAGTCCACGAAG R: TGTCACACATGGCAGGATCT	JX469587
Mpu B112w ^5^	fam	161–165	(AG)14	F: CCATTACAAGTGCTTGGAGACA R: TGGAACATGCTGGAAATTGT	JX469588
Mpu B202w ^3^	pet	164–168	(CTC)5(GA)14	F: TCTCCTCCTCCTCCTCCTTC R: ATGAGATTGACCGTGCATCA	JX469589
Mpu B209w ^2^	vic	119–125	(CT)16	F: TGCTTCTCCCTCTGACTGCT R: CCGCCCAAGTATCCCTAAAT	JX469590
Mpu B217w ^3^	vic	126–144	(TC)5(TC)17	F: TTCCCTGCTTGTGCTCTCTT R: TGGGGTAAGGGTAGGTATGC	JX469591
Mpu C4w ^7^	ned	147–163	(TCCA)9	F: CTGGCCCTATCACATACATATTCA R: GGAAGTATACTCATGCCTGCAA	JX469592
Mpu C102w ^3^	fam	119–135	(TGGA)7	F: GGGTGGATGGGTGAGTAGGTA R: CCTTCCCACATTCCATCCTT	JX469593
Mpu D207w ^5^	ned	188–214	(CT)10 (ATAG)7	F: CAGGTGAAGAAGTCCCTCTGT R: CTTGGTTCTGACCATTTGGA	JX469594
Mpu D209w ^4^	fam	165–185	(TATC)7	F: GAACAGCAAGTAGTCCAACTCTCA R: GTTGGATCCTTTCCATCACC	JX469595
Mpu D231w ^2^	fam	132–160	(GATA)9	F: TTTGGGTTCCACAGTAGGTG R: ATGCTCTCAATCCATGCTCA	JX469596

**Table 2:** Diversity calculations in samples of domestic ferret (*Mustela putorius furo*) from Australia and USA. A third set of samples contained European polecats (*Mustela putorius*, *N* = 6) and hybrids of *Mustela putorius furo* plus one of the following at 50% or more in reported pedigree: *Mustela putorius* (*N* = 4), *Mustela eversmanni* (*N* = 4), *and Mustela siberica* (*N* = 1). *N*_A_, number of alleles; *H*_O_, observed heterozygosity; and *H*_E_, expected heterozygosity.

Locus	Australia (*n* = 25)			USA (*n* = 25)			Polecat/Hybrid (*n* = 15)		
		
	*N*_A_	*H*_O_	*H*_E_	*N*_A_	*H*_O_	*H*_E_	*N*_A_	*H*_O_	*H*_E_
MpuA4w	2	0.16	0.15	4	0.64	0.59	8	0.80	0.75
MpuA10w	2	0.12	0.12	2	0.48	0.50	3	0.20	0.19
MpuA115w	2	0.60	0.50	1	0	0	6	0.40	0.41
MpuA121w	2	0.60	0.46	2	0.36	0.39	5	0.47	0.49
MpuA129w	2	0.16	0.22	1	0	0	3	0.27	0.25
MpuA212w	1	0	0	2	0.36	0.30	3	0.13	0.13
MpuA223w	3	0.76	0.58	2	0.32	0.27	7	0.67	0.81
MpuA229w	2	0.44	0.35	2	0.08	0.08	4	0.47	0.67
MpuA231w	2	0.60	0.50	2	0.28	0.30	6	0.73	0.73
MpuB1w	1	0	0	3	0.52	0.58	6	0.53	0.78
MpuB6w	3	0.56	0.65	3	0.32	0.34	5	0.53	0.70
MpuB9w	5	0.40	0.49	2	0.12	0.12	6	0.60	0.65
MpuB12w2	2	0.40	0.49	2	0.32	0.44	3	0.67	0.54
MpuB112w2	2	0.44	0.35	3	0.68	0.64	3	0.53	0.69
MpuB202w2	2	0.60	0.51	2	0.48	0.49	3	0.40	0.48
MpuB209w	3	0.52	0.58	3	0.36	0.36	4	0.47	0.61
MpuB217w	3	0.32	0.29	2	0.12	0.12	6	0.40	0.55
MpuC4w2	2	0.36	0.39	2	0.04	0.04	4	0.53	0.53
MpuC102w	1	0	0	1	0	0	4	0.33	0.36
MpuD207w	3	0.32	0.37	2	0.48	0.49	6	0.33	0.67
MpuD209w	3	0.64	0.51	2	0.36	0.35	4	0.47	0.58
MpuD231w2	2	0.20	0.25	4	0.40	0.45	6	0.80	0.76
Mean	2.3	0.37	0.35	2.2	0.31	0.31	4.8	0.49	0.56
